# Supplementary material for: Microglia-astrocyte crosstalk regulates synapse remodeling via Wnt signaling
Source: bioRxiv. 2024 Feb 9:2024.02.08.579178. Preprint. [Version 1] doi: 10.1101/2024.02.08.579178 (PMC10871360; doi:10.1101/2024.02.08.579178)
Supplement: Supplement 1 [file NIHPP2024.02.08.579178v1-supplement-1.pdf]

Supplementary Figure 1: Microglia synapse engulfment is increased and astrocyte process density is reduced after whisker lesioning

bioRxiv preprint doi: https://doi.org/10.1101/2024.02.08.579178; this version posted February 9, 2024. The copyright holder for this preprint (which was not certified by peer review) is the author/funder, who has granted bioRxiv a license to display the preprint in perpetuity. It is made available under aCC-BY 4.0 International license.

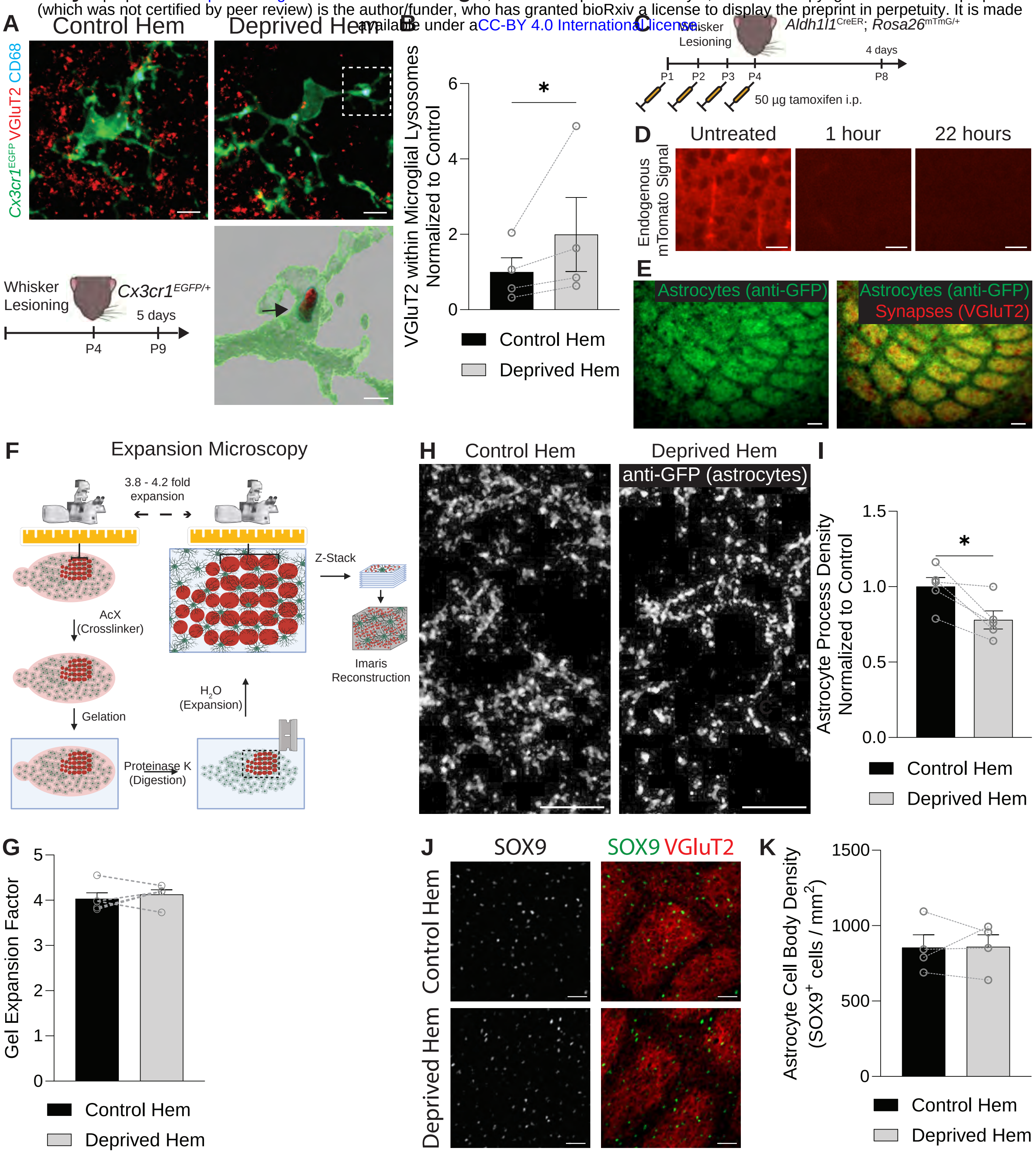

bioRxiv preprint doi: https://doi.org/10.1101/2024.02.08.579178; this version posted February 9, 2024. The copyright holder for this preprint (which was not certified by peer review) is the author/funder, who has granted bioRxiv a license to display the preprint in perpetuity. It is made available under aCC-BY 4.0 International license.

Figure S1. Microglial synapse engulfment is increased and astrocyte process density is reduced after whisker lesioning

Related to Figure 1

(A) Top: representative fluorescent max intensity projection images of anti-VGluT2$^+$ thalamocortical synaptic terminals (red), anti-CD68$^+$ lysosomes (cyan), and *Cx3cr1^{EGFP+}* microglia immunolabeled with anti-GFP (green) in layer IV of the barrel cortex in the control and deprived hemispheres at 5 days post-lesioning. Scale bars 5 µm. Bottom left: diagram of experimental protocol used to assess microglial engulfment of thalamocortical synaptic terminals in *Cx3cr1$^{EGFP/+}$* mice at 5 days after unilateral whisker lesioning. Bottom right: Imaris surface reconstruction of anti-VGluT2$^+$ synaptic material (red) contained within anti-CD68$^+$ lysosomes (cyan) inside *Cx3cr1$^{EGFP/+}$* microglia immunolabeled with anti-GFP (green) from the white outlined region. Arrow indicates an example of VGluT2$^+$ material contained within CD68$^+$ microglial lysosomes. Scale bar 2 µm.

(B) Quantification of anti-VGluT2$^+$ synaptic material within microglial lysosomes at 5 days after whisker lesioning shows more engulfed anti-VGluT2$^+$ material in the deprived hemisphere (hem) of the barrel cortex compared to the control hem (Ratio paired t test: n = 4 mice. * $p < 0.05$).

(C) Diagram of experimental protocol used to assess astrocyte-synapse contacts by expansion microscopy in *Aldh1l1^{CreER}; Rosa26$^{mTmG/+}$* mice at 4 days after unilateral whisker lesioning.

(D) Representative fluorescent images of endogenous mTomato signal in untreated brain sections from *Aldh1l1^{CreER}; Rosa26$^{mTmG/+}$* mice and after treatment with liberate antibody binding (LAB) solution. The endogenous mTomato signal is no longer detectable after 22 hours of incubation in LAB solution. Scale bars 20 µm.

bioRxiv preprint doi: https://doi.org/10.1101/2024.02.08.579178; this version posted February 9, 2024. The copyright holder for this preprint (which was not certified by peer review) is the author/funder, who has granted bioRxiv a license to display the preprint in perpetuity. It is made available under aCC-BY 4.0 International license.

(E) Representative fluorescent images of layer IV barrel cortex brain sections from *Aldh1l1*$^{CreER/+}$; *Rosa26*$^{mTmG/+}$ mice collected 4 days after unilateral whisker lesioning. Sections from the control and deprived hemispheres are labeled with anti-GFP to identify astrocyte processes and anti-VGluT2 to label thalamocortical synaptic terminals. Scale bars 20 µm.

(F) Diagram of experimental protocol to perform expansion microscopy. First, pre-expansion images are taken of brain sections from *Aldh1l1*^{CreER}*; Rosa26*$^{mTmG/+}$ mice immunolabeled with anti-GFP to detect astrocytes and anti-VGluT2 to detect thalamortical synaptic terminals in the barrel cortex. Sections are then incubated with the crosslinker Acryloyl-X SE (AcX), embedded in a gel, digested with proteinase K, cut down to the region of interest, and incubated in H_2O to isotopically expand the gel by ~3.8 - 4.2 times its original size. A post-expansion image is taken and measurements between specific landmarks are compared with the pre-expansion image to determine the gel expansion factor for each gel. Confocal z-stack images are taken for surface reconstructions using Imaris software.

(G) Quantification of the gel expansion factor in the control and deprived hems of *Aldh1l1*^{CreER}*; Rosa26*$^{mTmG/+}$ mice shows no differences between hems (Ratio paired t test: *n* = 5 mice).

(H) Representative fluorescent images of expanded layer IV barrel cortex brain sections from *Aldh1l1*$^{CreER/+}$; *Rosa26*$^{mTmG/+}$ mice collected 4 days after unilateral whisker lesioning. Sections from the control and deprived hem are labeled with anti-GFP to identify astrocyte processes. Scale bars 5 µm (corrected for expansion index).

(I) Quantification of astrocyte process density in layer IV of the barrel cortex at 4 days after whisker lesioning shows reduced density in the deprived hem compared to the control hem (Ratio paired t test: *n* = 5 mice. * $p < 0.05$)

bioRxiv preprint doi: https://doi.org/10.1101/2024.02.08.579178; this version posted February 9, 2024. The copyright holder for this preprint (which was not certified by peer review) is the author/funder, who has granted bioRxiv a license to display the preprint in perpetuity. It is made available under aCC-BY 4.0 International license.

(J) Representative fluorescent images from brain sections containing layer IV of the barrel cortex from the deprived and control hem of *Aldh1l1*^{CreER}; *Rosa26*$^{mTmG/+}$ mice at 4 days after whisker lesioning. Sections were immunolabeled with anti-SOX9 to label astrocyte nuclei and anti-VGluT2 to label thalamocortical synaptic terminals. Scale bars 50 µm.

(K) Quantification of the density of astrocytes (anti-SOX9^{+} cells) in layer IV of the barrel cortex at 4 days after whisker lesioning does not show a difference between the control and deprived hems (Ratio paired t test: *n* = 4 mice).

All data are presented as mean ± SEM.

Supplementary Figure 2: Enrichment of cell-type specific mRNA by TRAP-Seq

bioRxiv preprint doi: https://doi.org/10.1101/2024.02.08.579178; this version posted February 9, 2024. The copyright holder for this preprint (which was not certified by peer review) is the author/funder, who has granted bioRxiv a license to display the preprint in perpetuity. It is made available under aCC-BY 4.0 International license.

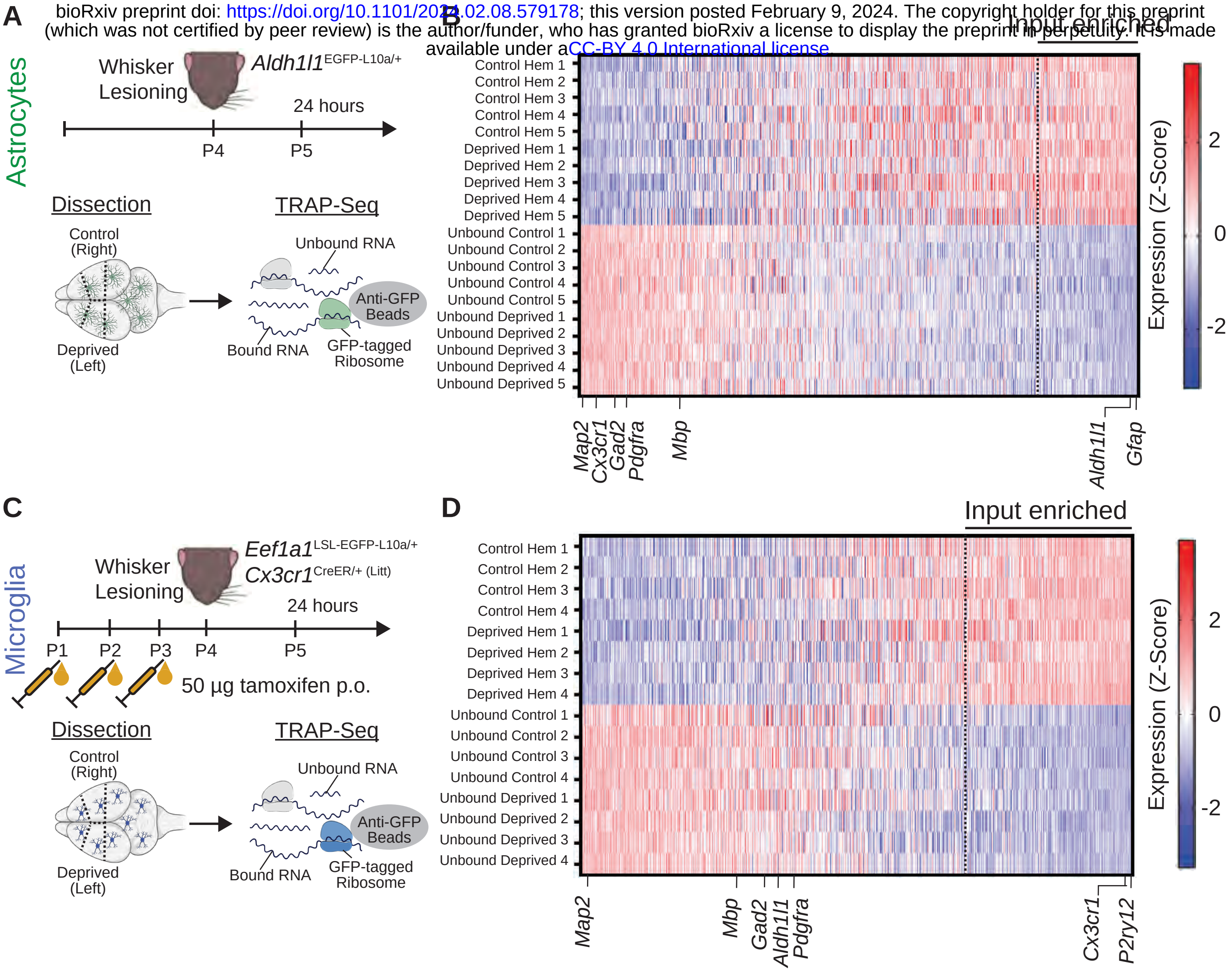

bioRxiv preprint doi: https://doi.org/10.1101/2024.02.08.579178; this version posted February 9, 2024. The copyright holder for this preprint (which was not certified by peer review) is the author/funder, who has granted bioRxiv a license to display the preprint in perpetuity. It is made available under aCC-BY 4.0 International license.

Figure S2. Enrichment of cell-type specific mRNA by TRAP-Seq

Related to Figure 3

(A, C) Diagram of experimental protocol used for TRAP-Seq analysis of ribosome-bound RNA from (A) astrocytes and (C) microglia isolated from the control and deprived hemispheres (hem) of the primary somatosensory cortex at 24 hours after unilateral whisker lesioning.

(B, D) Heatmaps showing expression levels of all genes detected in (B) astrocyte and (D) microglia TRAP-Seq experiments, sorted by enrichment in TRAP-enriched samples compared to unbound samples. Rows correspond to individual TRAP-enriched and unbound samples from control and deprived hems. Dashed black lines indicate the cutoffs used to determine input-enriched genes for downstream analyses for astrocytes (p-value < 0.05, |fold change| > 1.2, and mean expression > 5) and microglia (p-value < 0.05, |fold change| > 2, and mean expression > 5). Cell-type specific markers for astrocytes (*Aldh1l1*, *Gfap*), microglia (*Cx3cr1*, *P2ry12*), neurons (*Map2*), interneurons (*Gad2*), oligodendrocytes (*Mbp*), and OPCs (*Pdgfra*) are indicated. Only astrocyte cell-type markers are input-enriched in (B) and only microglia cell-type markers are input-enriched in (D), confirming that TRAP-isolations successfully enriched for the cell type of interest.

Supplementary Figure 3: Ablation of *Wls* in WLS cKO mice

bioRxiv preprint doi: https://doi.org/10.1101/2024.02.08.579178; this version posted February 9, 2024. The copyright holder for this preprint (which was not certified by peer review) is the author/funder, who has granted bioRxiv a license to display the preprint in perpetuity. It is made available under aCC-BY 4.0 International license.

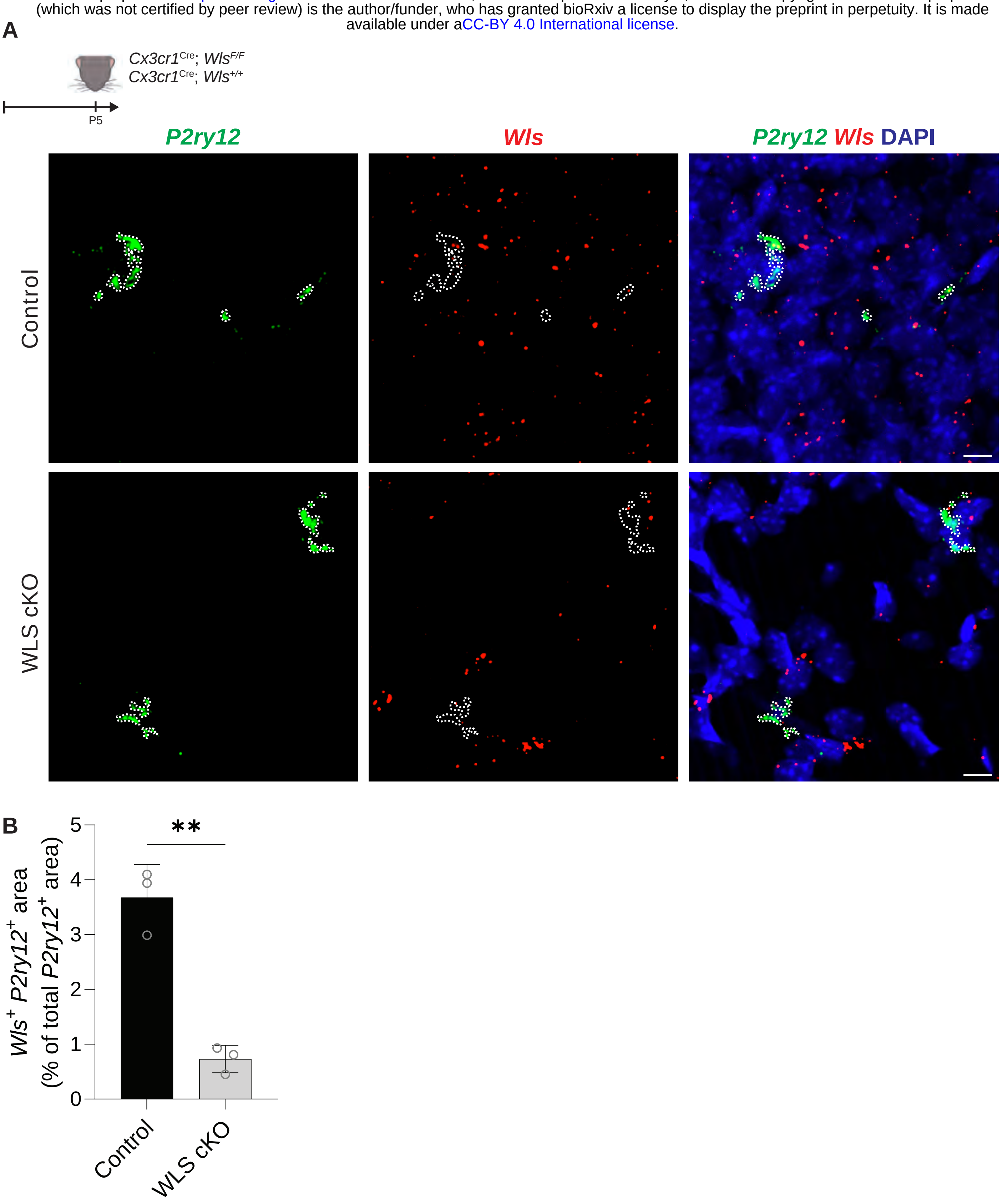

bioRxiv preprint doi: https://doi.org/10.1101/2024.02.08.579178; this version posted February 9, 2024. The copyright holder for this preprint (which was not certified by peer review) is the author/funder, who has granted bioRxiv a license to display the preprint in perpetuity. It is made available under aCC-BY 4.0 International license.

Figure S3. Ablation of *Wls* in WLS cKO mice.

Related to Figure 5

(A) (Top) diagram of experimental protocol used to perform *in situ* hybridization of *Wls* in $Cx3cr1^{Cre}$; $Wls^{F/F}$ (WLS cKO) and $Cx3cr1^{Cre}$; $Wls^{F/F}$ (control) mice at postnatal day 5 (P5). (Bottom) representative fluorescent images of brain sections from WLS cKO and control mice with *in situ* hybridization of *Wls* (red) and the microglia marker *P2ry12* (green). Nuclei are labeled with DAPI (blue). White outlines indicate the $P2ry12^{+}$ area. Scale bars 10 µm.

(B) Quantification of the percentage of $P2ry12^{+}$ area that overlaps with *Wls* fluorescence in WLS cKO mice and control mice at P5 shows reduced Wls^{+} area in WLS conditional mice, confirming that *Wls* was successfully ablated (Student's t test: n = 3 WLS cKO mice, 3 control mice. ** $p < 0.01$).

All data are presented as mean ± SEM.

Supplementary Figure 4: A pharmacological inhibitor of Wnt receptor signaling prevents synapse loss after whisker lesioning

bioRxiv preprint doi: https://doi.org/10.1101/2024.02.08.579178; this version posted February 9, 2024. The copyright holder for this preprint (which was not certified by peer review) is the author/funder, who has granted bioRxiv a license to display the preprint in perpetuity. It is made available under aCC-BY 4.0 International license.

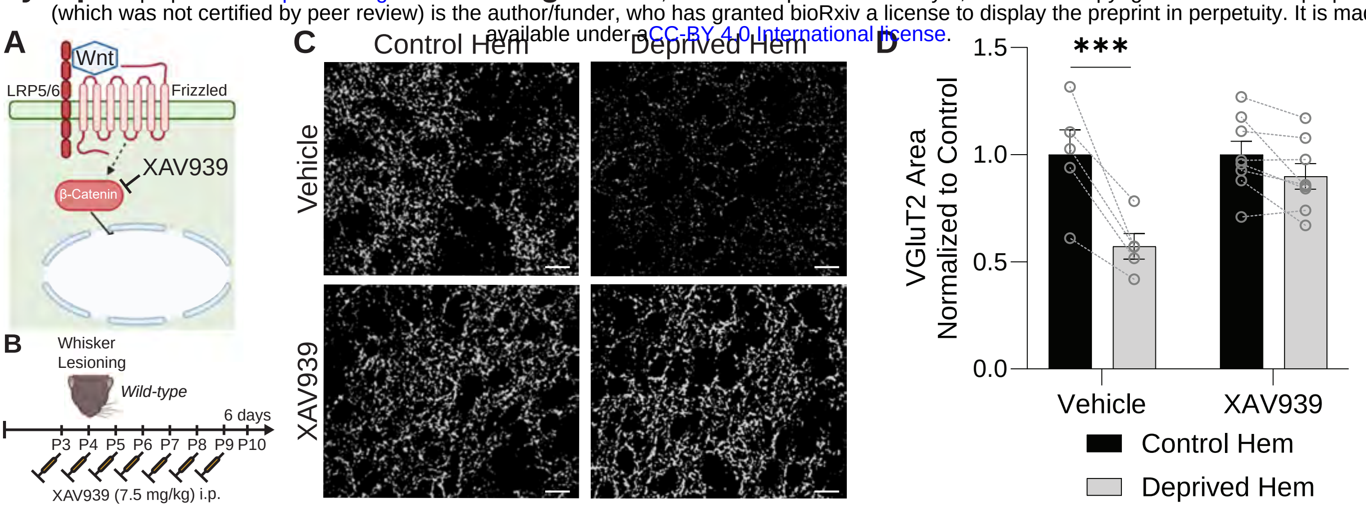

bioRxiv preprint doi: https://doi.org/10.1101/2024.02.08.579178; this version posted February 9, 2024. The copyright holder for this preprint (which was not certified by peer review) is the author/funder, who has granted bioRxiv a license to display the preprint in perpetuity. It is made available under aCC-BY 4.0 International license.

Figure S4. A pharmacological inhibitor of Wnt receptor signaling prevents synapse loss after whisker lesioning

Related to Figure 5

(A) Diagram of the effect of XAV939 on the canonical Wnt siganling pathway. XAV939 promotes the degradation of β-catenin, which prevents it from accumulating in the nucleus after binding of Wnt ligand to frizzled and LRP5/6 receptors.

(B) Diagram of experimental protocol used to assess the density of VGluT2$^+$ synaptic terminals in layer IV of the barrel cortex at 6 days after whisker lesioning in wild-type mice injected with the Wnt signaling inhibitor XAV939.

(C) Representative fluorescent images of anti-VGluT2$^+$ thalamocortical synaptic terminals in layer IV of the barrel cortex in the control and deprived hemispheres (hem) at 6 days post-lesioning in wild-type mice injected with XAV939 or vehicle. Scale bars 10 µm.

(D) Quantification of the density of anti-VGluT2$^+$ thalamocortical synaptic terminals in layer IV of the barrel cortex at 6 days post-lesioning shows reduced density in the deprived hem compared to the control hem in vehicle treated mice, but not in mice treated with XAV939 (Repeated measures 2-way ANOVA with Sidak's post hoc test: n = 5 vehicle mice, 8 XAV939 mice. *** $p < 0.001$).

All data are presented as mean ± SEM.

Supplementary Figure 5: Ablation of microglial Wnt release blocks reductions in astrocyte process density after whisker lesioning

bioRxiv preprint doi: https://doi.org/10.1101/2024.02.08.579178; this version posted February 9, 2024. The copyright holder for this preprint (which was not certified by peer review) is the author/funder, who has granted bioRxiv a license to display the preprint in perpetuity. It is made available under aCC-BY 4.0 International license.

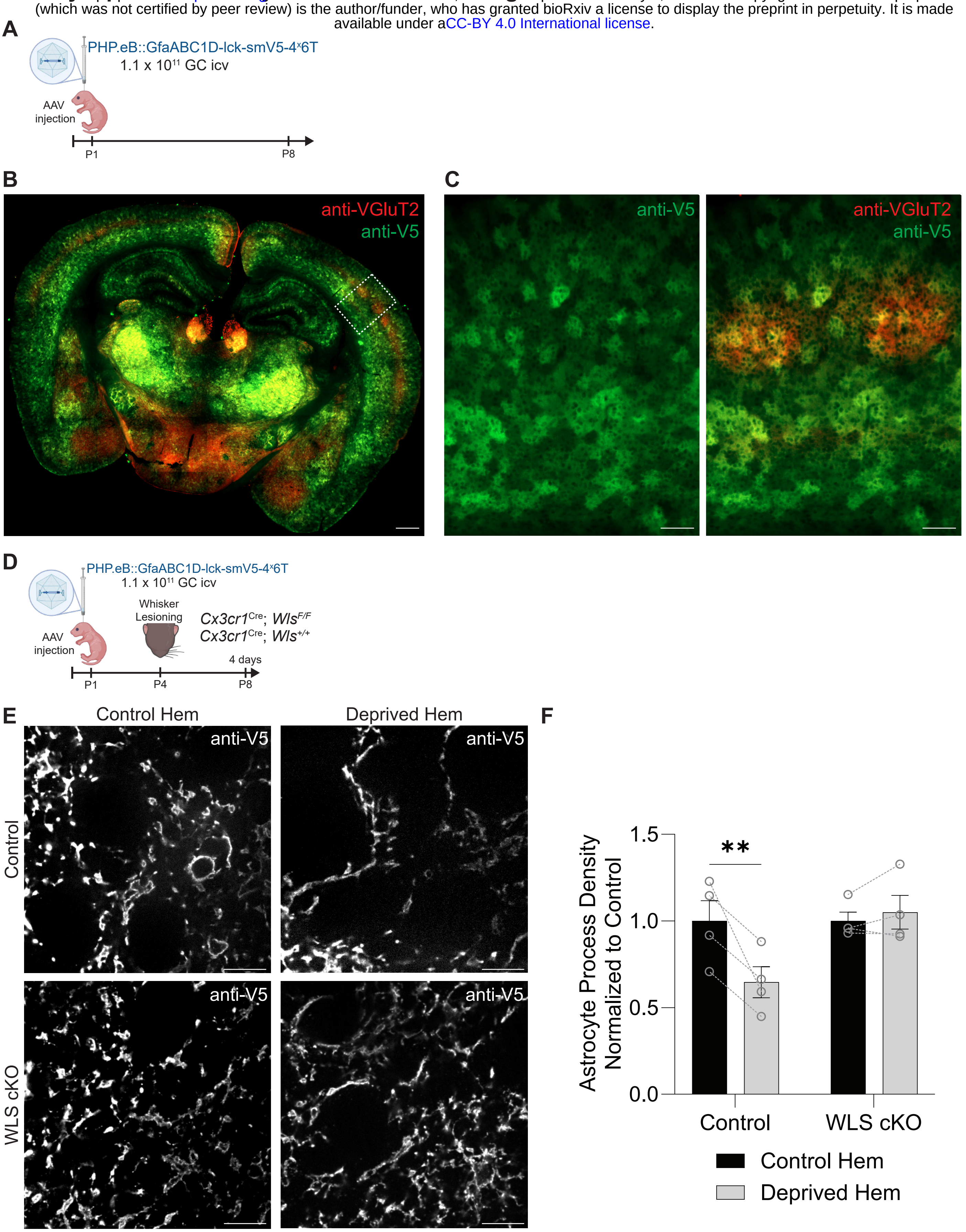

bioRxiv preprint doi: https://doi.org/10.1101/2024.02.08.579178; this version posted February 9, 2024. The copyright holder for this preprint (which was not certified by peer review) is the author/funder, who has granted bioRxiv a license to display the preprint in perpetuity. It is made available under aCC-BY 4.0 International license.

Figure S5. Ablation of microglial Wnt release blocks reductions in astrocyte process density after whisker lesioning

Related to Figure 5

(A) Diagram of experimental protocol used to label astrocytes with membrane-bound V5 protein at postnatal day 8 (P8) by intracerebroventricular (icv) injection of PHP.EB::GfaABC1D-lck-smV5-4^x6T adeno-associated virus (AAV).

(B-C) Representative fluorescent images of brain sections from P8 mice injected with PHP.EB::GfaABC1D-lck-smV5-4^x6T AAV, immunolabeled with anti-VGluT2 (red) and anti-V5 (green). White box region in (B) is shown at higher magnification in (C). Scale bars (B) 500 µm and (C) 100 µm.

(D) Diagram of experimental protocol used to assess astrocyte-synapse interactions in *Cx3cr1*^{Cre}*; Wls*$^{F/F}$ (WLS cKO) and *Cx3cr1*^{Cre}*; Wls*$^{F/F}$ (control) mice at 4 days after unilateral whisker lesioning. Astrocytes were labeled with membrane-bound V5 protein by icv injection of PHP.EB::GfaABC1D-lck-smV5-4^x6T AAV at P1.

(E) Representative fluorescent images of expanded layer IV barrel cortex brain sections from WLS cKO and control mice collected 4 days after unilateral whisker lesioning. Sections from the control and deprived hemispheres are labeled with anti-V5 to identify astrocyte processes. Scale bars 5 µm (corrected for expansion index).

(F) Quantification of astrocyte process density in layer IV of the barrel cortex at 4 days after unilateral whisker lesioning shows reduced process density in the deprived hem of control mice compared to the control hem, but not in WLS cKO mice (Repeated measures 2-way ANOVA with Sidak's post-hoc test: n = 4 control mice, 4 WLS cKO mice. ** $p < 0.01$).

All data are presented as mean ± SEM.
